# De novo transcriptome sequencing and SSR markers development for *Cedrela balansae* C.DC., a native tree species of northwest Argentina

**DOI:** 10.1371/journal.pone.0203768

**Published:** 2018-12-07

**Authors:** Susana L. Torales, Máximo Rivarola, Sergio Gonzalez, María Virginia Inza, María F. Pomponio, Paula Fernández, Cintia V. Acuña, Noga Zelener, Luis Fornés, H. Esteban Hopp, Norma B. Paniego, Susana N. Marcucci Poltri

**Affiliations:** 1 Instituto de Recursos Biológicos, Instituto Nacional de Tecnología Agropecuaria, Hurlingham, Buenos Aires Argentina; 2 Instituto de Biotecnología, Instituto Nacional de Tecnología Agropecuaria, Hurlingham, Buenos Aires, Argentina; 3 CONICET, Buenos Aires, Argentina; 4 Estación Experimental Agropecuaria, Instituto Nacional de Tecnología Agropecuaria, Famaillá, Tucumán, Argentina; 5 Facultad de Ciencias Exactas y Naturales, Universidad de Buenos Aires, Buenos Aires, Argentina; Youngstown State University, UNITED STATES

## Abstract

The endangered *Cedrela balansae* C.DC. (Meliaceae) is a high-value timber species with great potential for forest plantations that inhabits the tropical forests in Northwestern Argentina.Research on this species is scarce because of the limited genetic and genomic information available. Here, we explored the transcriptome of *C*. *balansae* using 454 GS FLX Titanium next-generation sequencing (NGS) technology. Following *de novo* assembling, we identified 27,111 non-redundant unigenes longer than 200 bp, and considered these transcripts for further downstream analysis. The functional annotation was performed searching the 27,111 unigenes against the NR-Protein and the Interproscan databases. This analysis revealed 26,977 genes with homology in at least one of the Database analyzed. Furthermore, 7,774 unigenes in 142 different active biological pathways in *C*. *balansae* were identified with the KEGG database. Moreover, after in silico analyses, we detected 2,663 simple sequence repeats (SSRs) markers. A subset of 70 SSRs related to important “stress tolerance” traits based on functional annotation evidence, were selected for wet PCR-validation in *C*. *balansae* and other *Cedrela* species inhabiting in northwest and northeast of Argentina (*C*. *fissilis*, *C*. *saltensis and C*. *angustifolia*). Successful transferability was between 77% and 93% and thanks to this study, 32 polymorphic functional SSRs for all analyzed *Cedrela* species are now available. The gene catalog and molecular markers obtained here represent a starting point for further research, which will assist genetic breeding programs in the *Cedrela* genus and will contribute to identifying key populations for its preservation.

## Introduction

The Meliaceae is a widely distributed family of trees mostly growing in rain forests of tropic and sub-tropic regions, with some species extending to seasonally dry forests and mangroves [1–5].

The *Cedrela* genus spreads in Central and South America and reaches its southernmost edge in a subtropical montane rainforest ecosystem known as the Yungas [6, 7], in the Northwestern Argentina (NWA). The Yungas provide environmental services such as watershed protection, the restoration of soil fertility and the stability of river basins and represents one of the sites of highest biodiversity in Argentina. *Cedrela* is among the most important forest resources; however, its heavy exploitation led to a depletion process of member of this genus, such as *Cedrela balansae* C.DC (Orán Cedar). This species has excellent wood properties and inhabits between 300 to 700 meters above sea level (masl) in the Piedmont Rainforest, within a small latitudinal range (22° to 24°30’S). Its habitat in Argentina has been modified by anthropogenic intervention [8, 9]. Therefore, its preservation and the enhancement of this species in the remaining forests is vital.

Several groups have studied the genetic diversity of *C*. *balansae* before using fragment length polymorphism (AFLP) and simple sequence repeats (SSRs) transferred from other species of the Meliaceae family [10–12].

As there is a domestic market demand for this wood, breeding for superior trees of this species should result in regional development and discourages withdrawals of the native forest. The importance of *Cedrela* to forestry lies in its speed for grow but biotic and abiotic factors, mainly low and freezing temperatures and shootborers, are the main constrains to establish commercial plantations of the *Cedrela* species particularly in the early years of growth.

Knowledge of the genetic bases of resistance to abiotic stress tolerance and insects are important factors for deciding the breeding strategies for genetic improvement of this species. Therefore, one of the major challenges for *Cedrela spp* tree cultivation is the selection of genotypes adapted to low and freezing temperatures, drought stress and tolerant to mahogany shoot borer. Such challenges could be confronted by developing new strategies and tools in the field of biotechnology. Next Generation Sequencing (NGS) technologies and Bioinformatic work-flows can easily identify SSR markers which are needed in population’ genetics and association studies. Several transcriptomic studies on tree species provided important information to discover genes of interest and new molecular markers, for example in *Quercus* spp [13, 14], *Pinus contorta* [15], *Nothofagus nervosa* [16], *Prosopis alba* [17] and *Pinus pinaster* [18] inter alia. In addition, the application of NGS approaches allowed new reports in members of the Meliaceae family, such as *Khaya Senegalensis* ([19], *Azadirachta indica* [20, 21], *Carapa guianensis* [22] and *A*. *indica* and *Melia azedarach* [23]. Nevertheless, little genomic information of the *Cedrela* genus from Argentina is available to date.

In this study, we provide a reference transcriptome for *C*. *balansae* and identify SSR markers to support diversity studies in wild populations. This information also contributes with tools for the genetic breeding programs, such as the programs of INTA (National Institute of Agricultural Technology of Argentina) that are focused on improving the behavior of seedlings.

## Materials and methods

### Plant material

RNA extraction was performed with leaves from four seedlings of cold tolerant families, whose mothers come from San Andrés and Pintascayos populations (Argentinian Yungas). Leaves were collected from 6-month seedlings under spring season with a temperature range between 10–20°C. The samples seeds were collected on private lands with the owner permission.

For SSR (70) wet validation, we used DNA of eight *C*. *balansae* individuals from seven origins (Apolinario Saravia, San Andrés, Rio Seco Forestal Santa Bárbara, Rio Seco Familia Falcón, Piquirenda, Calilegua National Park, Yuto).

This sampling covers most of the natural distribution area of this species in Argentina. The characterization of 11 polymorphic SSRs was performed with 51 individuals from three native populations of the natural distribution area.

The transferability of 70 SSRs from *C*. *balansae* (source species) to *C*. *fissilis*, *C*. *angustifolia* and *C*. *saltensis* (target species) was evaluated by assessing leaf samples of eight individuals of either eight populations from *C*. *fissilis*, seven from *C*. *angustifolia* and three from *C*. *saltensis*. The sampling represents the whole range of the species.

### RNA isolation and sequencing

RNA extraction was performed according to Chang’s protocol [24] that is specific to woody plants. Briefly, 1 g of fresh tissue was grounded to a fine powder by using liquid nitrogen. Then, two extractions were performed with chloroform and RNA was precipitated with LiCl_2_, extracted for a second time with chloroform and finally precipitated with ethanol. The obtained RNA was resuspended in 50 μl of DEPC (diethylpyrocarbonate) treated water and was quantified using a Nanodrop 1,000 spectrophotometer. The quality was assessed with a 2,100 Bioanalyzer (Agilent Technologies Inc.). Total RNA was purified using Poly (A) Purist kit (Ambion) and the quality was assessed again with a 2,100 Bioanalyzer (Agilent Technologies). cDNA was synthesized using cDNA Kit (Roche) for constructing a shotgun library. Roche 454 GS FLX Titanium sequencing platform at INDEAR (Rosario Agro Biotechnology Institute, http://webservices.indear.com/) in Rosario, Argentina performed sequencing. Moreover, all bioinformatic analysis was performed in the Bioinformatics Unit at the Biotechnology Institute at INTA.

### Transcript assembly and analysis

Raw data were preprocessed discarding low-quality reads and the resulting high-quality cleaned reads were assembled de novo into contigs, isotigs and isogroups using Newbler Assembler Software 2.6 p1 (Roche, IN, USA).

The reads identified as singletons (i.e., reads not assembled into isotigs) after assembly were subjected to CD-HIT-454 clustering algorithm using a sequence identity cut-off of 90%, which eliminates redundant sequences or artificial duplicates.

The assembled sequences were compared against an in-house Viridiplantae non-redundant protein database (NCBI-NR) using BLASTX with a cutoff E-value of 1e^-10^ [25]. Annotation and mapping routines were run with BLAST2GO, which assigns Gene Ontology annotation [26], (www.geneontology.org/), KEGG maps (Kyoto Encyclopedia of Genes and Genomes, KASS) and an enzyme classification number (EC number) using a combination of similarity searches and statistical analysis [27]. In addition to BLAST2GO, the full suite of InterProScan [28] was run with default parameters to extend the functional annotation and GO term assignment by means of protein signature recognition methods.

#### SSR identification

To identify SSRs for all possible combinations of dinucleotide, trinucleotide, tetranucleotide and pentanucleotide repeats, we used the SSR webserver (GDR) (https://www.rosaceae.org). This webserver uses the GETORF algorithm (EMBOSS Package) and selects the longest ORF as the putative coding region. This webserver also uses Primer 3 (v.0.4.0) [29, 30] to design primer pairs. The criterion used for the SSR selection based on the minimum number of repeats was as follows: five for dinucleotide, four for trinucleotide, three for tetra, penta and hexanucleotide motives. The locations of specific SSR motifs within predicted UTRs and coding sequence regions were also analyzed.

#### SSRs validation, characterization and transferability

PCR reactions consisted of 5 ng of total DNA, 0.25 μM of each primer, 3.0–4.5 mM of MgCl_2_, 0.25 mM of each dNTP, 1X of PCR buffer and 0.6 U of Taq polymerase (Inbio). The PCR reactions consisted of a denaturation step of 2 min at 94°C followed by 28 cycles at the touchdown temperature of 51 or 56° (45 s at 92°C, 45 s at 51 or 56° and 45 s at 72°C) and the final extension step at 72°C for 10 min. Some microsatellites required a single temperature ranging from 55–62°C. The amplification products were separated on a 6% (w/v) denaturing polyacrylamide gel and were stained using the DNA silver staining procedure of Promega (USA) following the manufacturer’s instructions. The SSR profiles were scored manually for each of the SSR loci. The molecular weight of each band was estimated in base pairs (bp) by comparison with a 10 bp DNA Ladder (Invitrogen) using the GEL software (Dubcovsky J., unpublished data) based on the reciprocals method [31].

Polymorphic amplification patterns (P), compatible with the species ploidy level in the expected size within or out of range and monomorphic (M) patterns of all the markers were defined. M corresponded to a single band of equal molecular weight.

The DNA used here for all studies had previously been extracted and used in earlier works [12, 32].

#### Usefulness of SSRs marker for diversity analysis

To assess the potential of the 11 novel SSRs for genetic diversity analysis, we evaluated 51 individuals of *C*.*balansae*. Total number of alleles (Na), observed (Ho) and expected heterozygosity or gene diversity per locus (He) were calculated running the GenAlEx 6.5 program [33] in https://biology-assets.anu.edu.au/GenAlEx/Download.html. The Polymorphism Information Content (PIC) was estimated with Microsatellite Toolkit [34].

Also, the estimation of linkage disequilibrium (DL) to account for a non-independent segregation of markers [35] and Inbreeding coefficients (Fis) related to consanguineous mating were computed through the GDA 1.1 program [36].

## Results

The sequencing run resulted in 212,589 single end reads, with an average of 434 bp (approximately 92.3 Mbp) that represent 1.2 X of *Cedrela odorata* genome [37]. After filtering for adaptors, primers and low-quality sequences, we obtained 202,010 high quality sequences for assembly analysis (95% of raw sequences). Because no reference genome sequence was available for *Cedrela species*, all the filtered high-quality reads using the Newbler assembly Software v. 2.6 (Roche, IN, USA) were assembled. The assembly process resulted in the identification of 2,620 contiguous sequences (contigs) and 32,117 singletons. Contig sequences were further assembled into 1,531 isotigs which are the putative transcripts re-constructed from the contig data. Moreover, to distinguish the transcripts that come from the same locus, we set the Newbler software to group all isotigs that shared a common contig-graph. This run resulted in 1,266 isogroups, which could be considered as putative “loci”. In addition, CD-HIT-454 algorithm was used to eliminate artificial duplicates when clustering all singletons. After clustering, we detected 25,580 (80%) unique singletons longer than 200 bp. Table 1 displays an overview of the sequencing and assembly process.

Most isogroups (84.4%) had only one isotig, whereas the others contained between 2 and 13 isotigs. The average of transcripts per locus was 1.2.

**Table 1:** Overview of the sequencing and assembly of *C*.*balansae* leaf transcriptome.

Description	Statistics
Total number of raw read sequences	212,589
Mean length (bp)	434
Total Number of assembled read sequences	149,572
Total number of isotigs (>200 bp)	1,531
Average length of isotigs (>200 bp)	976.7
Range of isotig length (>200 bp)	214–9,135
Total number of singletons (>200 bp)	25,580
Average length of singletons (bp)	424.7
Range of singleton length (bp)	200–728
Total number of unigenes	27,111

The sum of all isotigs and singletons, hereon named unigenes, longer than 200 bp (27,111 unigenes) were considered for further downstream analysis.

**Fig 1:**
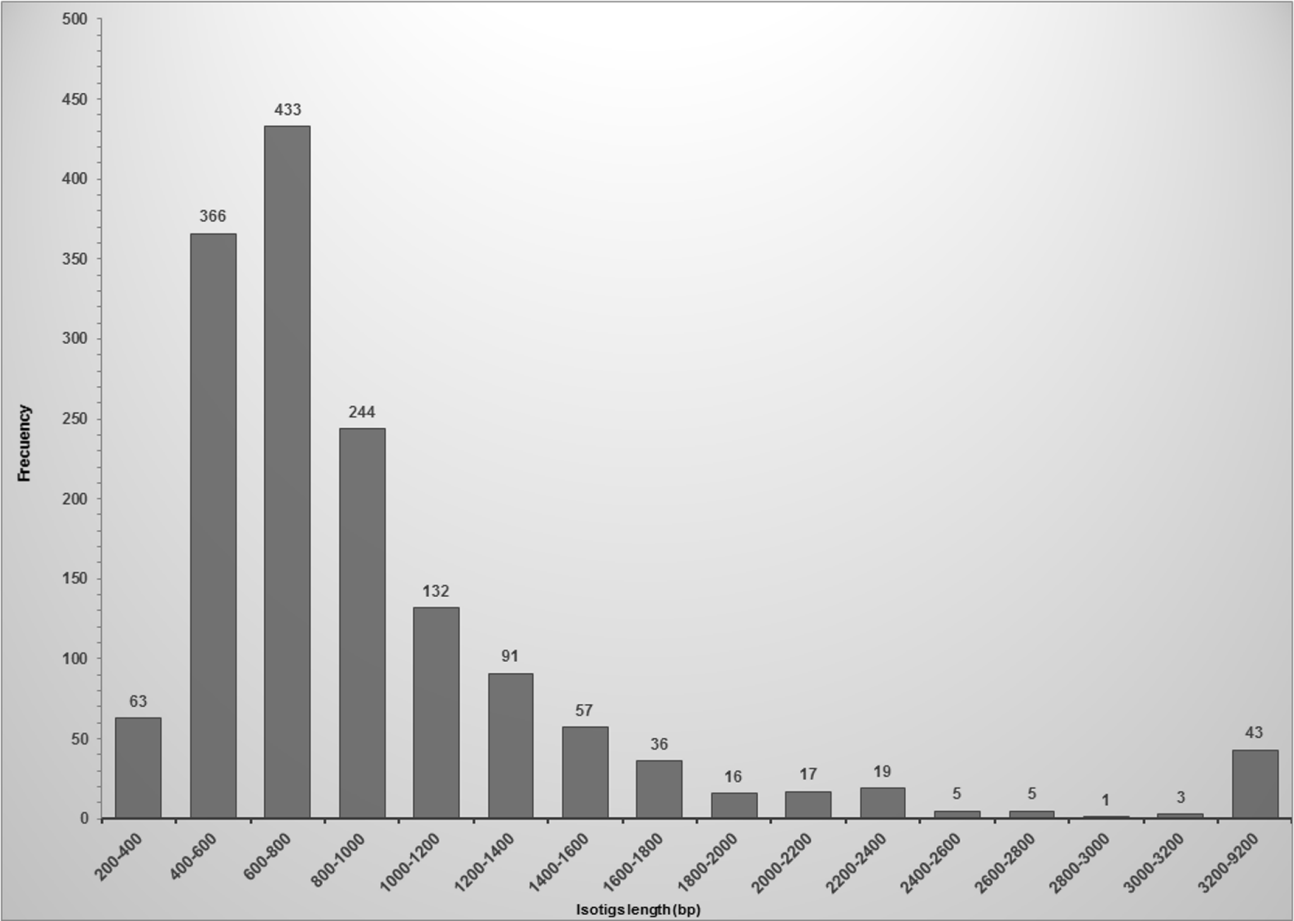
Frequency distribution of isotigs length. The histograms represent the number of isotig sequences in relation to their length.

The size distribution of isotigs ranged from 214 to 9,135 bp, with an average of 976.7 bp and an N50 equal to 1,033 bp (Fig 1). More than 90% of the isotigs were between 200 to 1,500 bp and 50% of the assembled bases grouped into isotigs greater than 744 bp. The coverage depth for isotigs ranged from 1 to 6 contigs.

The singleton length ranged from 200 to 728 bp, with a mean of 424.7 (Table 1) and most of them (69%) were between 400 bp and 600 bp (data not shown).

For the functional annotation, 27,111 unigenes were searched in the nr-Protein from NCBI using BLASTx tool. From this analysis, 20,953 sequences (77.3%) had at least one positive hit. When we analyzed the unigenes with the Interproscan suite, data annotation reached 99.65% after scanning all databases in the suite (Table 2).

**Table 2:** Summary of functional annotation of assembled *C*. *balansae* unigenes.

Database	Tools	# of annotated transcripts	% of annotated transcripts
Nr Protein	BLASTx	20,953	77.33
InterProScan	Full interpro suite	26,977	99.65
GO	BLAST2GO	19,029	64.00
KEGG	BLAST2GO	7,774	29.00

This Transcriptome Shotgun Assembly project has been deposited at NCBI TSA database within BioProject: PRJNA451202. Moreover, all raw data (single end reads) were deposited in the NCBI SRA database with run name: SRR7050098 and sample name SAMN08964270 (https://www.ncbi.nlm.nih.gov/nuccore/GGMM00000000).

Approximately 99% of unigenes longer than 1,000 bp, depicted matches with genes of the databases and only 54% of the unigenes shorter than 300 bp showed suitable matches (S1 Fig).

**Fig 2:**
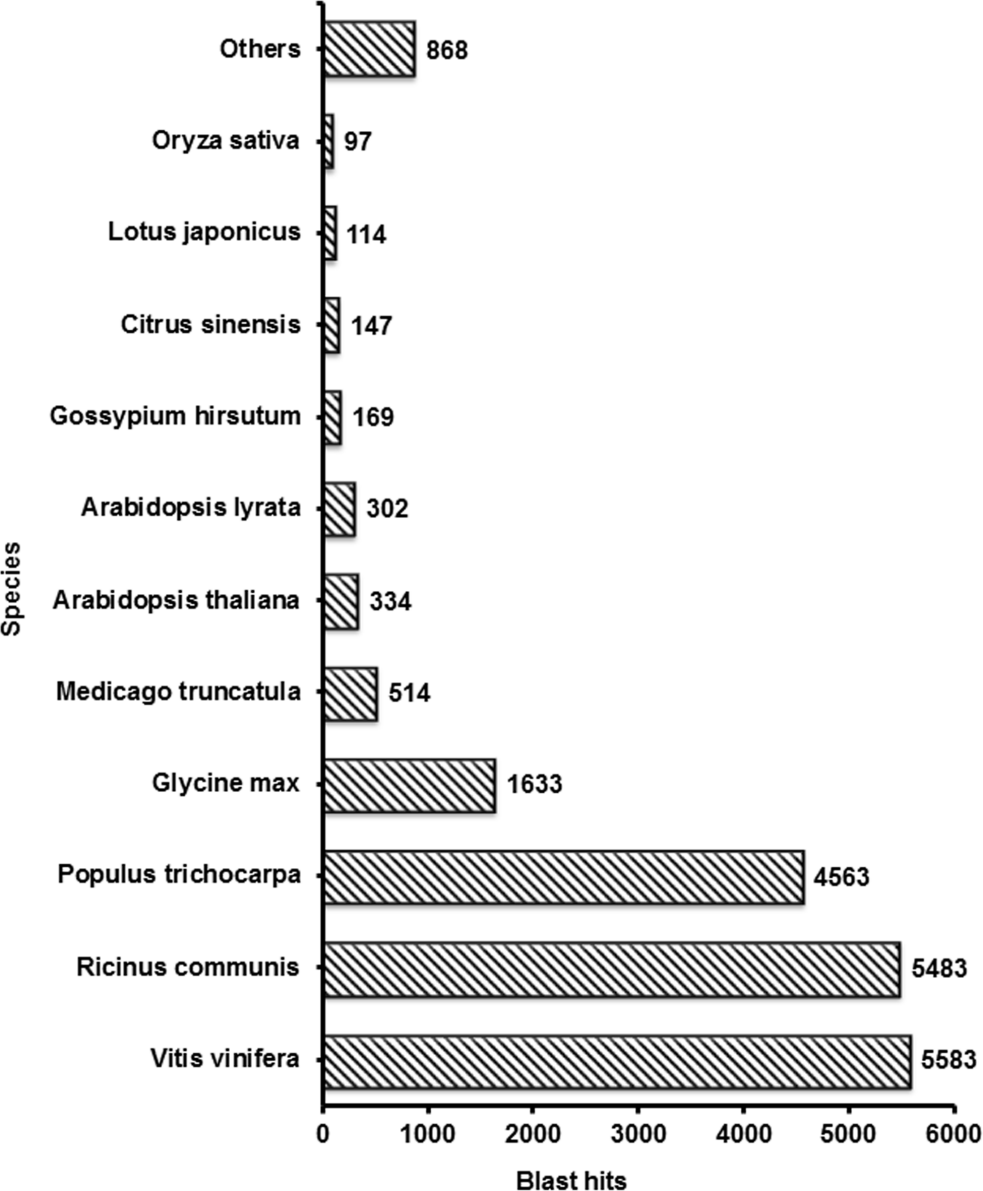
Summary of functional annotation of assembled *C*.*balansae* unigenes. Gene Ontology terms were assigned successfully to 19,029 of the BLASTX annotated unigenes using BLAST2GO. These unigenes were classified in three main groups: biological process, cellular components and molecular functions. For biological processes, the most represented GO term was cellular process followed by metabolic process and response to stimulus. For cellular components, genes associated with cell parts and organelles were the most highly represented, while genes related to binding and catalytic activity represented the largest proportion of genes with molecular functions. Fig 3 shows more information on the functional categorization.

*C*. *balansae* unigenes showed the highest homology with the semi-woody plant *Vitis vinifera*, (28%), followed by the woody plants *Ricinus communis* (27.6%) and *Populus trichocarpa* (23.0%) (Fig 2).

**Fig 3:**
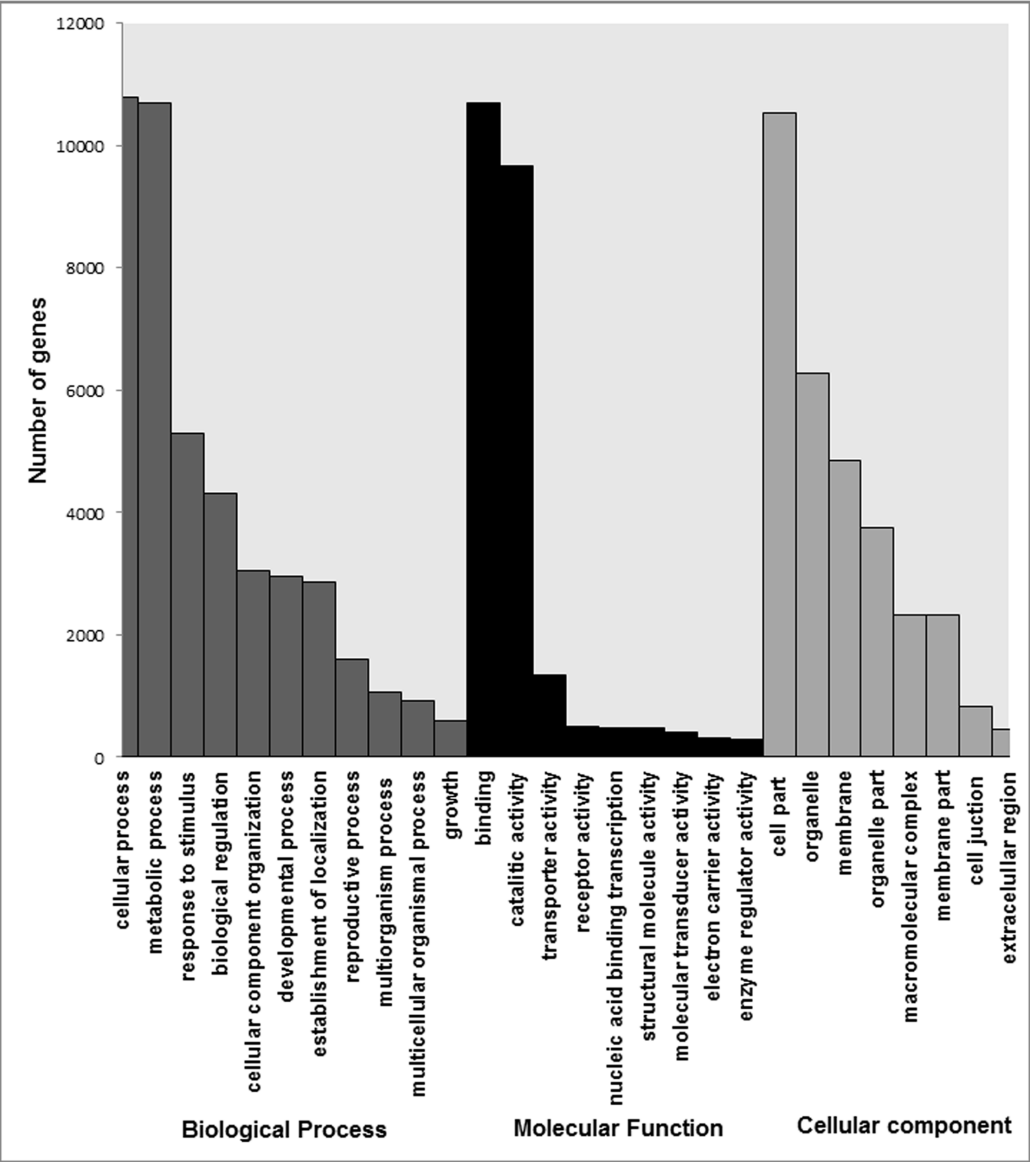
Gene ontology (GO) classification of annotated *C*. *balansae* unigenes.

Total 10,548 unigenes, were assigned Enzyme Comission (EC) numbers and the most represented enzyme classes were transferases, hydrolases and oxidoreductases (S2 Fig).

All unigenes were contrasted against the KEGG database to identify the active biological pathways in *C*. *balansae* and 7,774 unigenes were mapped to 142 different KEGG pathways. The type classification of pathways from the KEGG annotation results indicated genes related to metabolism representing the largest proportion, especially carbohydrate metabolism, lipid metabolism and amino acid metabolism. Purine metabolism was the second largest category in the classification (S1 Table).

**Fig 4:**
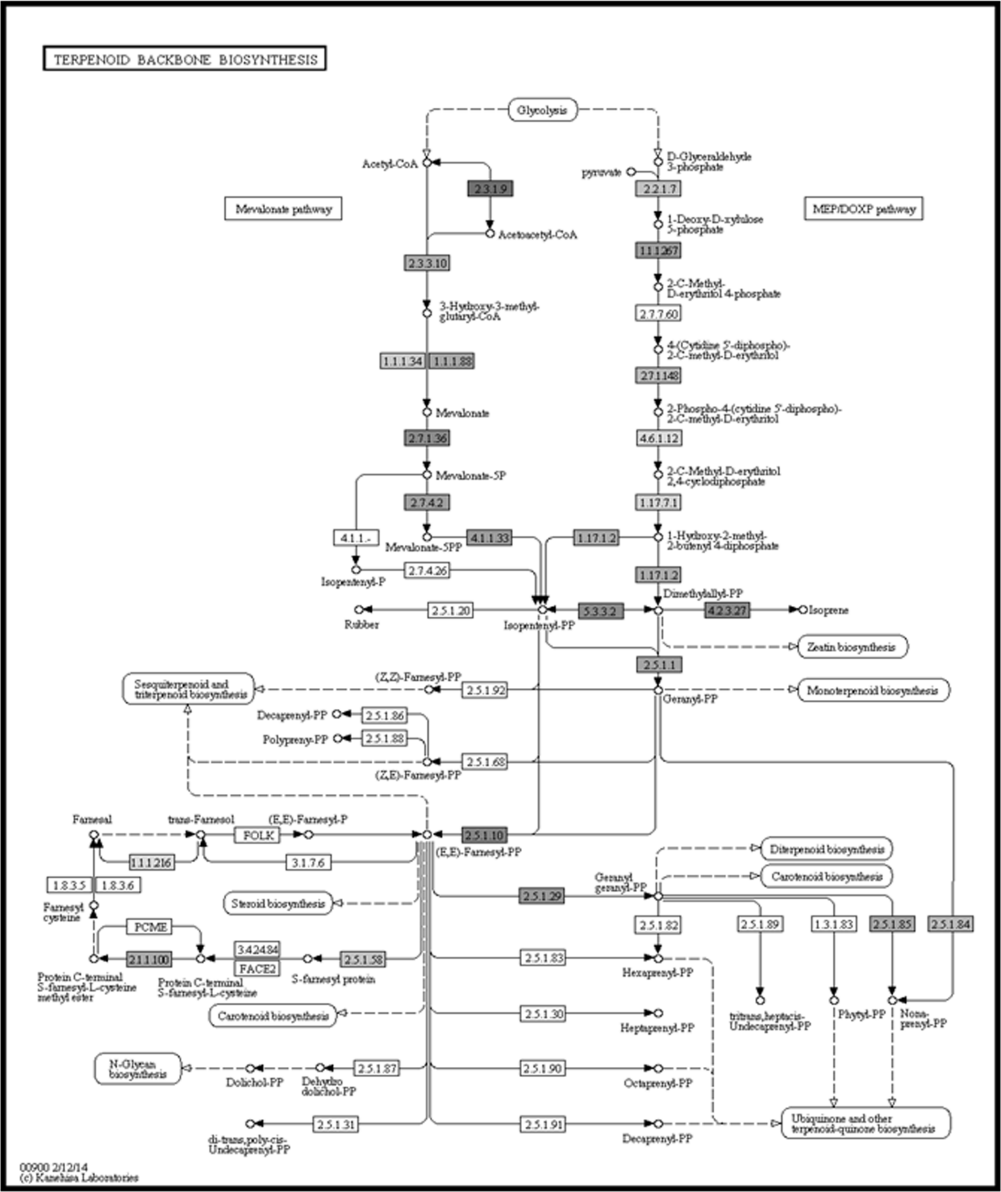
The terpenoid biosynthesis pathway. Color boxes indicate the identified genes in *C*. *balansae* transcriptome.

Regarding secondary metabolites, we identified 70 genes involved in the terpenoid backbone biosynthesis (Fig 4), 45 in ubiquinone and other terpenoid-quinone, 9 in diterpenoide biosynthesis, 7 in sesquiterpenoid and triterpenoid biosynthesis, and 6 in monoterpenoid genes.

### SSRs marker development and validation

Repeat motifs were analyzed to explore the SSR profiles in the *C*. *balansae* leaf transcriptome unigenes. The criterion used for the SSR selection was based on the minimum number of repeats (see Materials and Methods). Thus, we identified 2,663 SSRs (262 in isotigs, 2,401 in singletons) within 27,111 unigene sequences. When we considered multiple repeat occurrences in a same unique locus: 670 of the unigenes contained more than one SSR and 1,993 unigenes contained one SSR. Detailed information of the SSRs is described in S2 Table.

As expected, trinucleotide repeat motifs were the most frequent type of microsatellite (1,269; 48%) in this study. The other repeat motifs showed much lower frequencies, with 639 (24%) 588 (22%) and 167 (6%) for tetra, dimeric and pentanucleotide repeats respectively. In addition, 1,932 (72.5%) SSRs, 216 (11%) isotigs and 1,716 (89%) singletons had sufficient flanking sequences to allow the design of appropriate unique primers to generate PCR products within the range of 100–300 bp. About 1,133 (43%) of the SSR sequences were inside ORF sequences, being most of them tri-nucleotide repeats (63%). Other repeat types were much less frequent (di 17%, tetra 16% and penta 4%). In the UTRs region, most SSRs (66%) were tri-nucleotide and tetra-nucleotide motifs. Finally, we selected SSRs belonging to gene families associated with important traits to *Cedrela* genus, such as cold-hardiness, drought tolerance, growth rate, etc. to assist the selection of best genotypes in the early stages of breeding programs.

To validate the SSR developed, we identified a subset of 70 SSR loci based on its sequence length, GC content and mainly the functional annotation (S2 Table). Then, we select those related to the following categories: transcription factors (Zinc fingers, Myb), peroxidases, among other categories of which 51% were located in predicted ORF’s. We tested all loci for PCR amplification and polymorphism in eight genotypes belonging to the eight remaining populations of *C*. *balansae*. Sixty-three (90%) out of the 70 SSRs were effectively amplified thus validating the quality of assembly. Of these SSRs, 52 were monomorphic and 11 (16%) polymorphic. Although most SSR loci were monomorphic, some of them could show polymorphism in another sample of individuals. Furthermore, the putative biological function assigned to the validated and polymorphic SSR corresponded to response to cold (64%) and other stress stimulus (36%) (Table 3).

**Table 3:** Polymorphic SSRs primer pairs derived from *C*. *balansae* unigenes.

Locus name	Marker ID name	Motif	Primer sequence 5'-3'	Amplicon length expected	Sequence description
**TrCbal8**	isotig00700c	(gga)5	F:AAATTCCTTTCTTCTCCTTGGC	196	dehydrin 2 Vitis yeshanensis
			R:GAAAAGATTGACGACTTACCCG		
**TrCbal9**	isotig00766a	(ggc)7	F:CCAGAAAAATACCAGGAAGTGG	153	unnamed protein product Thellungiella halophila
			R:TTGAGTTTGAGCAGGAGTGCTA		
**TrCbal15**	isotig01103a	(ctgc)3	F:CAGGTCATTTCAGAAAGCTTCA	136	predicted protein Populus trichocarpa
			R:ATGACTAGAGATGGACCGCAAT		
**TrCbal16**	GR7D2IN01A9N43	(ggc)4	F:GTCGAGTTTGTGATCGAATCTG	262	Predicted glycine-rich protein 2-like Vitis vinifera
			R:CACCTCCTCCCTGATAACAATC		
**TrCbal27**	GR7D2IN02JVDNY	(aattt)3	F:AATGCCTCCAAGGATTAACAAG	201	predicted protein P. trichocarpa
			R:TTGGGTGATATTCAACTTGCAG		
**TrCbal38**	isotig00209b	(aga)5	F:TTTCTTCCCTCGAAGTAGGGTT	132	carbonic anhydrase, putative Ricinus communis
			R:CCACATTACGCCCTACTGTTTT		
**TrCbal42**	isotig00797a	(ta)5	F:GCATAACCAAACATACTGGGTG	208	Dehydration-responsive protein RD22 precursor
			R:AAGTGCTCAAAGTTAAGCCAGG		
**TrCbal43**	isotig00125a	(ta)6	F:ATTTGCTGCACTGAACACATTC	242	Inositol-3-phosphate synthase
			R:GCTAAGGAGAAAAGTGGATGGA		
**TrCbal47**	GR7D2IN01C6ECWa	(gac)5	F:TGCCTTAATCTCGTCTTCACAA	209	hypothetical protein ARALYDRAFT
			R:AACCTGATTCGCCTGAACTAGA		
**TrCbal61**	GR7D2IN01B4HGH	(ctc)5	F:TCCGATTATTCCGACAATCC	178	cold shock protein, putative Ricinus communis
			R:GGAAGTGGTGGTGCTTGTTT		
**TrCbal64**	GR7D2IN02HQKGO	(acat)3	F:TCAAGAATCACACACAGACGC	251	temperature-induced lipocalin P.tremuloides
			R:ACTGATCCACCACCAGAAGG		

Table 4 shows the results of the analyses performed with the 11 novel SSRs to assess their genetic information content in a larger sample of 51 accessions belonging to the *C*. *balansae* sampled from across the natural range of the species. Thus, we identified 30 allelic variants through the 11 polymorphic loci with 2 to 5 alleles per SSR. The He and PIC values ranged from 0.075 to 0.491 and from 0.073 to 0.370 for loci Trcbal 8 and Trcbal 47, respectively.

**Table 4:** Results of genotyping 51 *C*. *balansae* samples with 11 SSRs.

Locus name	Na	He	Ho	PIC	LD	Fis
TrCbal8	2	0.075	0.078	0.073	0.643	-0.045
TrCbal9	5	0.165	0.176	0.159	0.664	-0.052
TrCbal15	2	0.251	0.294	0.219	0.604	-0.171
TrCbal16	4	0.403	0.314	0.343	0.215	0.248
TrCbal27	4	0.076	0.078	0.075	0.660	-0.051
TrCbal38	2	0.128	0.137	0.120	0.613	-0.092
TrCbal42	2	0.251	0.255	0.219	0.570	-0.025
TrCbal43	2	0.095	0.100	0.090	0.718	-0.306
TrCbal47	2	0.491	0.471	0.370	0.357	0.040
TrCbal61	3	0.147	0.157	0.140	0.641	-0.127
TrCbal64	2	0.483	0.082	0.366	0.020	0.769

Na: number of alleles. Ho and He: observed and expected heterozygosity.PIC: polymorphism index content..LD: linkage desequilibrium Fis:estimated inbreeding coefficient.

Only one locus (TrCbal64) showed linkage disequilibrium (LD) and three (TrCbal64, TrCbal16, TrCbal43) had excess homozygotes (inbreeding) using as cutoff value: Fis ~ 0.200 (Table 4).

### Cross transferability of SSR marker to three *Cedrela* species

We successfully transferred 68 out of the 70 SSRs screened in *C*. *balansae* at least in one other target species. *C*. *fissilis*, *C*. *saltensis* and *C*. *angustifolia* showed higher cross-amplification level with 92.9%, 88.6% and 77.1% of transferability, respectively. In *C*. *fissilis*, we detected more polymorphic SSRs (27; (39%) than in *C*. *balansae* (16%), which is the source species, in *C*. *saltensis* (16%) and in *C*. *angustifolia* (9%). Among the target species, 31 were polymorphic (32 including *C*. *balansae*, the source specie).

In *C*. *fissilis*, the number of alleles ranged from 2 to 5, whereas in *C*. *saltensis* and *C*. *angustifolia*, it ranged from 2 to 4 and 2 to 5, respectively. The range of allele size revealed several loci showing variation among species. (S2 Table).

## Discussion

### Utility of NGS for gene and marker discovery in non-model species

The development of several new Next Generation Sequencing (NGS) techniques in the last decades can now generate large volumes of data, especially important for non-model organisms. Bioinformatics is a discipline of great expansion and development in recent years as a necessary complement to the implementation of large-scale genomics applied to the study of organism. NGS allowed us to generate a large fraction of *C*. *balansae* transcriptome. Indeed, *C*. *balansae* is a non-model species with scarce genomic information available in the literature prior to this study. So far, only 17 sequences for *C*. *balansae* are available in the Genbank, and no markers have been published.

### Marker identification and characterization

NGS provides an affluence of potentially useful markers that increase possibility of finding associations with functional genes and therefore with phenotypes. SSR markers derived from expressed sequences are faster to obtain, although they are considered less informative because DNA sequence is conserved in transcribed regions [38].

SSRs detected in this work require evaluation of their transferability and characterization for their usefulness for genetic diversity analyses among and within the *Cedrela* genus. In this study, we successfully amplified 97% SSRs of the analyzed SSRs (70) and 16% of these SSRs resulted polymorphic in *C*. *balansae*. Although the number of polymorphic SSRs detected in this study is low, another potential 299 polymorphic SSR (16% of total) from the 1,869 SSRs is expected.

Similar results were found in others native tree species, *Nothofagus* and *Prosopis*, where 20% and 15% of the tested SSRs respectively were polymorphic [16, 17]. A similar research with Illumina sequencing technology in sesame showed that about 90% primer pairs successfully amplified DNA fragments [39], which is comparable with our results (97%) and confirms the quality of sequencing and assembly here used. Moreover, the SSR frequency observed *in C*. *balansae* transcriptome was 9.8%, comparable to that reported in sugarcane (7.96%) [40], and also higher than in other *Meliaceae* species (3%) [19].

Furthermore, the presence of the trinucleotide motif that was observed as the most frequent in *C*. *balansae* was in consonance with the characteristics of the SSRs found in the transcriptomic sequences of *Nothofagus* nervosa and *Prosopis alba* tree species. [16, 17].

In the UTRs region, tri-nucleotide and tetra-nucleotide motifs were more frequent (66%); this value is slightly higher than that found in *Nothofagus spp*, which was 45% [18] and comparable to that detected in *Prosopis alba* [17]. Such dominance of triplets over other repeats in coding regions may be explained based on the selective disadvantage of non-trimeric SSR variants in coding regions, possibly causing frame-shift mutations [41].

The estimation of genetic diversity with these novel SSRs in 51 samples from three populations indicated low levels of polymorphism (He = 0.233; Ho = 0.195; PIC = 0.198 to 0.370) as expected in genic SSRs. A similar analysis using neutral SSRs showed moderate diversity genetic values [10, 12]. This difference could be attributed to the marker type used. In general, most transcriptome-derived loci detected lower level of polymorphism than that derived from genomic libraries [42–44]. Moreover, the moderate to low genetic diversity average values observed with both kind of markers is in accordance with the small latitudinal range of its distribution area in Argentina, as well as with the impact of the indiscriminate logging on the *C*. *balansae* species [8, 9, 10–12].

### Cross-transferability

In general, the transferability of functional markers to congeneric species seems to be a good approach to quickly obtain a set of compatible markers. [42, 45, 46]. According to this information, our results showed high levels of cross-amplification in the target species, *C*. *fissilis* (93%), and *C*. *saltensis* (89%). The transferability efficiency of the same set of markers slightly decreased to 77% in *C*. *angustifolia*, owing to the greater phylogenetic and morphological distance that exists between *C*. *angustifolia* and the other three species of the Cedrela genus [47–49].

In general, the mean transferability was higher (86%) than in the cross-amplification carried out with the same three target species of Meliaceae (43%) with neutral SSRs [50]. However, in this last study, the researchers assessed.other source species (*C*.*odorata*, *Swietenia humilis* and *Swietenia macrophylla*) [50], except for *C*. *fissilis* which was used in both studies. The high level of transferability of microsatellites from transcriptome sequences is attributable to a higher sequence conservation in primer binding sites [51]. Additionally, transferability also depends on the genetic distance between species.

Moreover, the 70 SSRs analyzed resulted in about 46% (32) polymorphic markers for all the studied species. Therefore, we could expect to identify at least other 859 potential polymorphic SSRs from a set of 1,869 markers that have not yet been analyzed in the *Cedrela* species.

## Conclusions

We performed a *de novo* transcriptome sequencing analysis of *C*. *balansae* leaf tissues using a 454-sequencing platform. This is the first report on the whole transcriptome of *Cedrela* genus. Genomic resources such as sequence of transcriptomes, genes and SSRs from this study will have a profound application to study diversity and traits association related to biotic and abiotic factors in *C*. *balansae* and other species of the *Meliaceae* family.

## Supporting information

S1 Table *Cedrela balansae* genes description. This table provides information on the annotation of isotigs and singletons, GO information and the enzymes putatively encoded by the RNA sequences, based on homology prediction and their associated pathways. This includes KEGG maps, enzyme names, and sequences ID.
(XLSX)

S2 Table SSRs derived of *Cedrela balansae* leaf transcriptome. List of SSR markers, *in vitro* polimorphic SSRs, Transferability of 70 SSRs.
(XLSX)

S1 Fig Effect of sequence length on the proportion of homology to models species. (TIFF)

S2 Fig Catalytic activity distribution in annotated *C*. *balansae* unigenes. (TIFF)
