# Supplementary figures and images for: De novo transcriptome sequencing and SSR markers development for Cedrela balansae C.DC., a native tree species of northwest Argentina
Source: PLoS One. 2018 Dec 7;13(12):e0203768. doi: 10.1371/journal.pone.0203768 (PMC6285271; doi:10.1371/journal.pone.0203768)

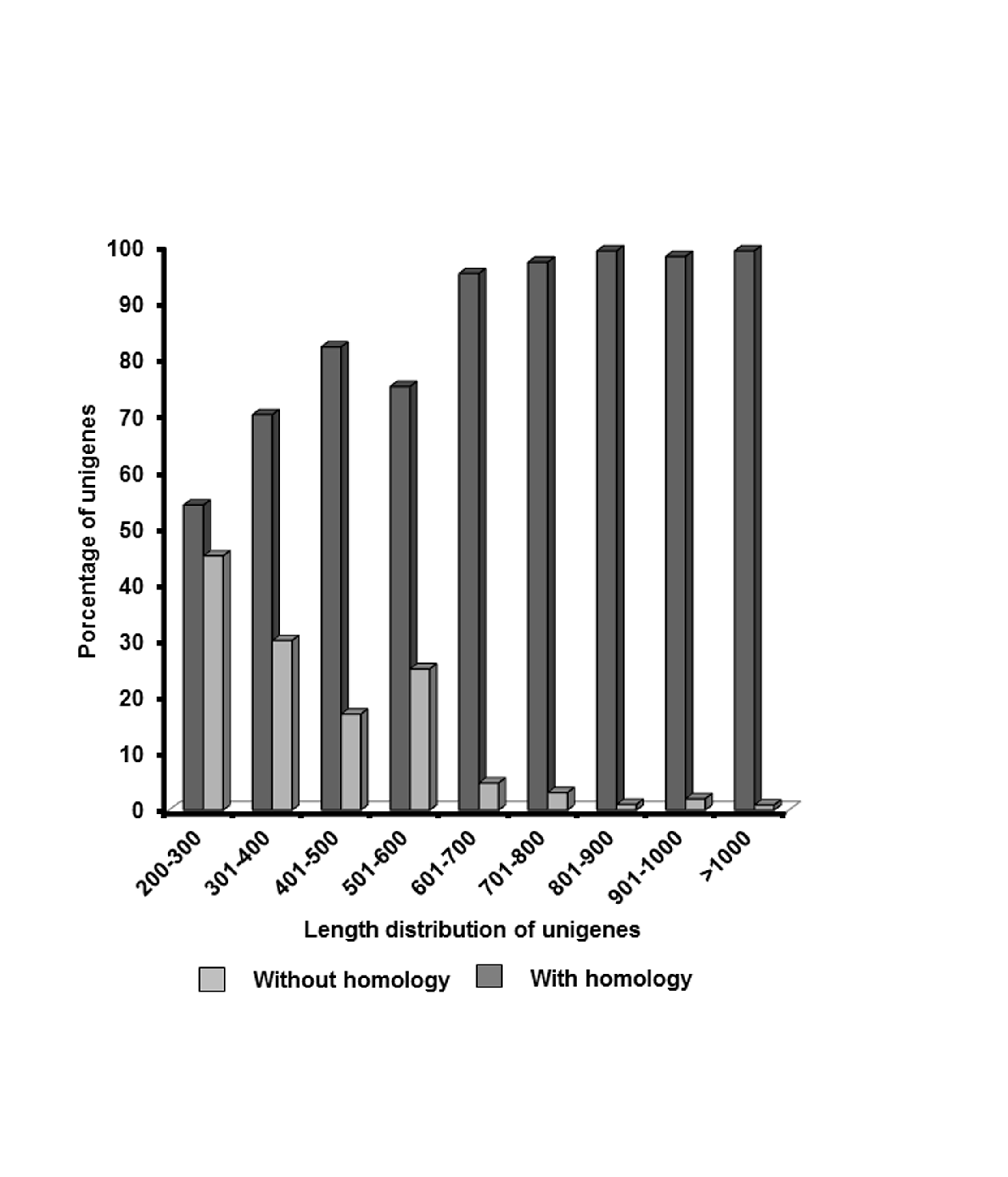

Supplement: S1 Fig — (TIFF) [file pone.0203768.s003.tiff]

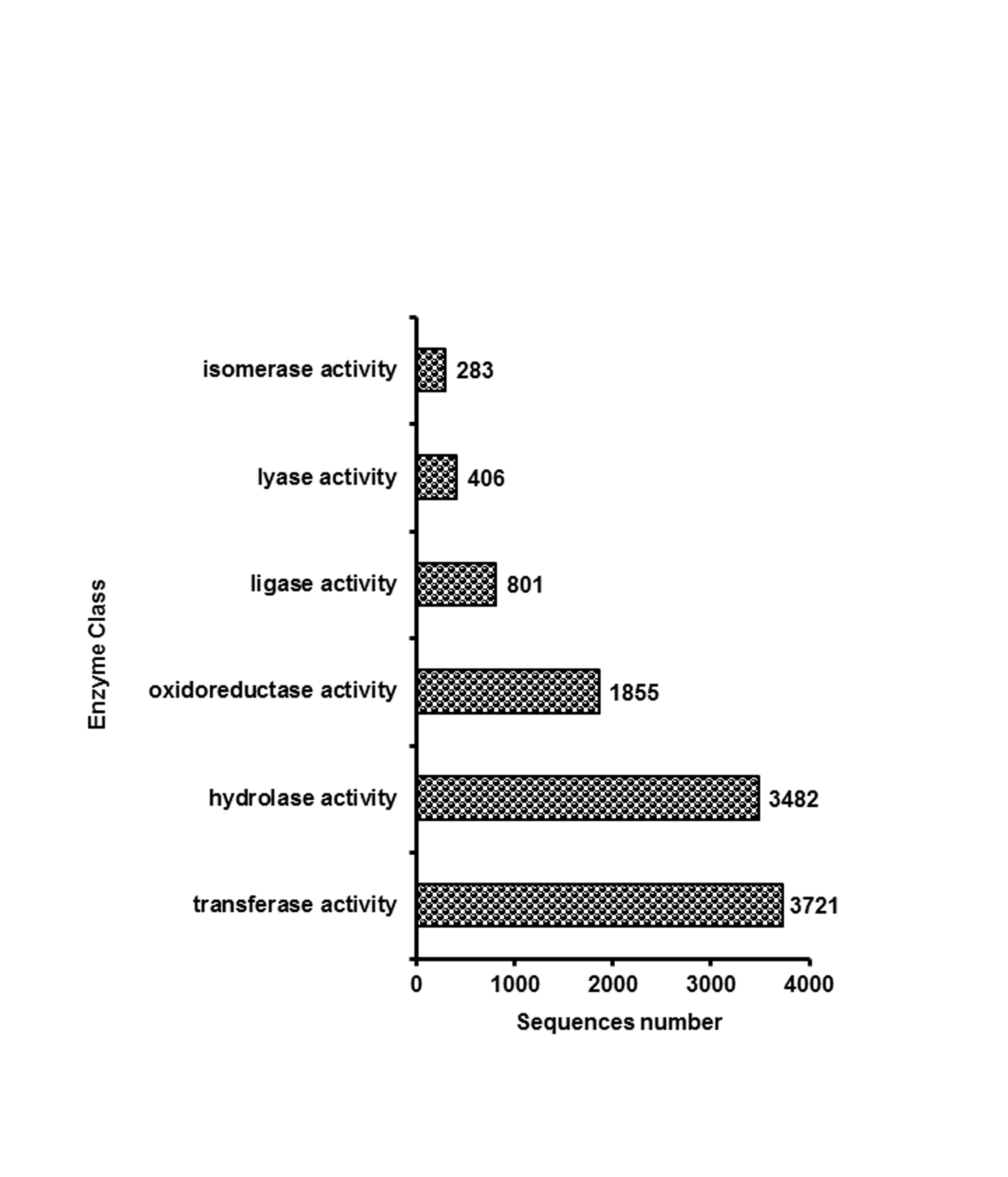

Supplement: S2 Fig — (TIFF) [file pone.0203768.s004.tiff]
